# The Microbiotic Highway to Health—New Perspective on Food Structure, Gut Microbiota, and Host Inflammation

**DOI:** 10.3390/nu10111590

**Published:** 2018-10-30

**Authors:** Nina Wærling Hansen, Anette Sams

**Affiliations:** 1 Molecular Endocrinology Unit (KMEB), Department of Endocrinology, Institute of Clinical Research, University of Southern Denmark, DK-5000 Odense, Denmark; nwhansen@health.sdu.dk; 2 Department of Clinical Experimental Research, Glostrup Research Institute, Copenhagen University Hospital, Nordstjernevej 42, DK-2600 Glostrup, Denmark

**Keywords:** carbohydrates, fiber, food structure, formulation, plant, microbiota, inflammation, metabolism, nutrition guidelines

## Abstract

This review provides evidence that not only the content of nutrients but indeed the structural organization of nutrients is a major determinant of human health. The gut microbiota provides nutrients for the host by digesting food structures otherwise indigestible by human enzymes, thereby simultaneously harvesting energy and delivering nutrients and metabolites for the nutritional and biological benefit of the host. Microbiota-derived nutrients, metabolites, and antigens promote the development and function of the host immune system both directly by activating cells of the adaptive and innate immune system and indirectly by sustaining release of monosaccharides, stimulating intestinal receptors and secreting gut hormones. Multiple indirect microbiota-dependent biological responses contribute to glucose homeostasis, which prevents hyperglycemia-induced inflammatory conditions. The composition and function of the gut microbiota vary between individuals and whereas dietary habits influence the gut microbiota, the gut microbiota influences both the nutritional and biological homeostasis of the host. A healthy gut microbiota requires the presence of beneficial microbiotic species as well as vital food structures to ensure appropriate feeding of the microbiota. This review focuses on the impact of plant-based food structures, the “fiber-encapsulated nutrient formulation”, and on the direct and indirect mechanisms by which the gut microbiota participate in host immune function.

## 1. Introduction

The gut microbiota is a complex ecosystem residing in the gastro-intestinal (GI) tract and consists of a diverse microbiotic community living in symbiosis with the host. A diverse microbiota is considered a major positive regulator of the interdependent metabolic and immune function of the host. The gut microbiota is shaped alongside the host immune system in a synergistic partnership [1]. Trillions of microorganisms participate in the maturation and regulation of immune function, energy metabolism, and hormonal balance [2], including regulation of intestinal mucosal barriers, fermentation of undigested nutrients, and synthesis of short chain fatty acids (SCFA) [3]. Furthermore, the microbiota releases microbiota-derived antigens [4,5], vitamins [6,7], and other molecules, such as tryptophan metabolites [8,9], that interact with the host biology. 

The dynamic relationship between the microbiotic ecosystem and its host is emphasized by the joint utilization of consumed nutrients. Diet is a shared substrate between the host and the gut microbiota and the choice of diet affects host health both directly and indirectly by affecting the abundance and composition of the microbiotic community [10,11].

The structural organization of complex carbohydrates determines the site of digestion and absorption in the GI tract [12], and hence the level of cross feeding [13], community organization, and proliferation of specific microorganisms. Importantly, all plant cell walls consist of complex carbohydrate structures that are digested selectively by microbiotic enzymes and not by host enzymes. Thus, complex polysaccharides in plant cell walls function as both substrate for the microbiota and as a sustained release delivery system of other plant-cell derived nutrients and biomolecules for both microbiotic and host utilization. The structural properties of the cell wall vary between different plant cells [14], and in theory determine the specific site of plant cell wall degradation and nutrient release.

Plant-based foods can be placed on a moving scale from whole foods (unrefined) to fully refined foods, and currently no official distinction based on the degrees of refinement exists. Foods with added refined nutrients (e.g., sugar, starch or oils) or any food, which has been structurally disrupted on the macroscopic level are considered refined. The degree of refinement is thus determined by the degree of macroscopic structural disruption (e.g., intact whole grain vs. ground whole grain vs. sifted flour) and the degree of refined supplementation. When plant-based food structures are completely disrupted requiring no microbiotic interaction for digestion we consider the food fully refined.

Microbiota composition varies greatly among different cultures and individuals [15,16], and specific genera of bacteria are more abundant in healthy individuals as compared to individuals in different disease states, as shown, for example, for *Bifidobacterium* and *Lactobacillus* [17,18,19], whose functions include carbohydrate fermentation and vitamin synthesis [6,20,21]. The degradation and utilization of complex carbohydrates rely on specific microbiotic enzymes secreted from the microbiota in the lower intestine [22]. The fermentation process yields energy for microbiotic proliferation and metabolites, e.g. SCFA [23] for regulation of inflammatory responses [24] and gut hormone secretion [25] in the host. The latter is of great importance for glucose homeostasis and plays a major role for the metabolic and inflammatory health of the host [26].

Microbiota composition is also closely connected to the integrity of our intestinal mucosal barrier. Upon microbiota dysbiosis mucosal bacteria can impair epithelial function and cause increased gut permeability with consequent immune dysregulation, leading to inflammatory disease states [27]. A number of disorders with an inflammatory component including obesity, inflammatory bowel diseases (IBD), type 2 diabetes (T2D), colorectal cancer, and cardiovascular diseases have been linked with dysbiosis [28,29,30,31,32]. This association presents the gut microbiota as a potentially modifiable factor in the etiology of these conditions. It is therefore of great importance for public and individual health to recognize that microbiota composition may be diversified within days or weeks upon introduction of unrefined plant-based diets [16].

In clinical trials diets are often classified as low carbohydrate vs. high carbohydrate, and low fiber vs. high fiber. These trials lack discrimination between refined and unrefined carbohydrates. Acknowledging the degree of refinement would likely strengthen the reproducibility of diet intervention studies for reasons discussed in the present review [33,34,35].

This paper analyzes the interplay between structures of plant-based foods or “fiber-encapsulated nutrients”, the gut microbiota, and their joint impact on host nutrient bioavailability, immune function, and metabolic function, and proposes a simple tool to select plant-based foods from a nutritional- and microbiota-promoting perspective. The review provides a biological explanation of the health superiority of structurally maintained and unrefined foods compared to nutrient-matched refined foods, combining classic and current scientific knowledge within nutrition, pharmaceutical science, microbiology, and plant biology. In addition, we add the perspective of “fiber as nutrient encapsulation” to the well-established molecular benefits of fiber as microbiotic nutrient (prebiotic). This structural perspective should be included in the current nutritional guidelines, which focus on nutrient and fiber content rather than nutrient and fiber structure.

## 2. Carbohydrates: Different Structures, Different Properties

The structural characteristics of dietary carbohydrates include the chemical composition, physicochemical properties, and resistance to human digestive enzymes. Dietary polysaccharides display a variety of linkages, branching, and polymerizations and must be broken down into their corresponding monosaccharides (e.g., glucose, galactose, and fructose) before they can be absorbed and metabolized by humans. This carbohydrate breakdown is initiated by α-amylase secreted by salivary glands, continues with pancreatic amylase in the duodenum, and is completed by intestinal enzymes (e.g., sucrase and lactase) located in the brush-border membrane of the enterocytes in the small intestine [22]. Pancreatic amylase hydrolyzes α-linked glucose polymers such as starch and glycogen, but humans lack the enzymes necessary to hydrolyze β-linked glucose polymers like those present in resistant starches and dietary fibers found in plant structures [36].

Resistant starches include starch where (i) the granules are inaccessible to enzymes due to conformation; (ii) the starch is retrograded or (iii) chemically modified. Importantly, starch may also be digestion-resistant due to encapsulation in plant cell fiber matrices [37] and new perspectives on this aspect are the main focus of this review.

Dietary fiber includes a large variety of carbohydrate polymers, e.g., xylans, β-glucans, fructans, β-mannans, celluloses, hemicelluloses, and pectins [38]. Cellulose, hemicellulose, and pectin are major components of plant cell walls, and starch [39] and inulin [40] are heavily represented in plant carbohydrate storage. As such, the above-mentioned molecular carbohydrates are regarded as prebiotics that may stimulate or alter the preferential growth of health-promoting bacterial species already residing in the colon [41].

The microbiota encodes a broad spectrum of enzymes catalyzing the depolymerization and further degradation of polysaccharides [22]. The structural properties of the carbohydrates are important since the ability to utilize different substrates differs between bacterial species, for example, it has been shown in vitro, that *Roseburia* spp. can utilize amylopectin starch and that *Bifidobacterium* spp. cannot [42].

Plant polysaccharides can be separated based on origin (cereals and grains, fruits, vegetables nuts, and legumes), and chemical compositions and physicochemical properties vary depending on origin [14]. The physicochemical properties of plant polysaccharides include fermentability, solubility, and viscosity, each of which impact site and degree of fermentation. Soluble fibers, such as short-chain fructo-oligosaccharides and pectin are depolymerized by bacterial enzymes more proximally in the GI tract, while fibers with a lower solubility, such as cellulose, are partially fermented in the distal colon where transit time is slower and microbiotic density higher [12].

Simple carbohydrates, such as monosaccharides, disaccharides, and oligosaccharides can be classified as host nutrition with direct utilization by host enzymes, whereas complex carbohydrate polymers, such as plant polysaccharides and resistant starches are vital substrates for the microbiota. Thus, a diet that includes a variety of unrefined plant structures can therefore promote proliferation of a broader spectrum of bacteria with specific intrinsic properties. A diet rich in plant structure variety also represents a diverse sustained release device for delivery of various nutrients and bioactive molecules for metabolic and immunological stimulation, as described below.

## 3. Plant Cell Structure

**Figure 1:**
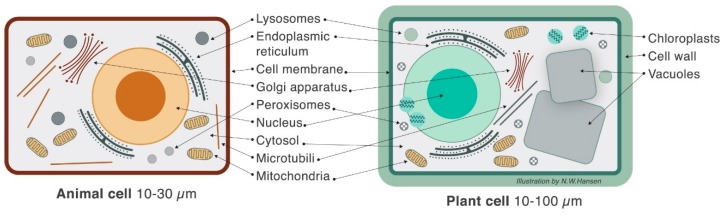
Schematic illustration of an animal and a plant cell. The cell wall (mainly complex carbohydrates), vacuoles (usually starch or lipid storage, depending on cell type), and chloroplasts are plant-cell specific structures that serve as nutrients for the microbiota, whereas the cellular content serves as signal molecules for host metabolic and immune regulation as well as nutrients for both host and microbiota. An intact plant cell is defined as unrefined food whereas the degree of structural disruption (and the supplementation of additives) defines the degree of refinement.

The plant cell wall is the major difference between animal and plant cells. Except for the cell wall, chloroplasts, and vacuoles, animal and plant cells share intracellular structures and compartmentalization (Figure 1).

The most characteristic component found in all plant cell walls is the fiber, cellulose [43], which consists of a collection of β-1,4-linked glucan chains that interact with each other via hydrogen bonds to form a crystalline microfibril [44]. In addition to cellulose, the primary plant cell wall contains two categories of matrix polysaccharides-pectins and hemicelluloses, and several proteins and glycoproteins, including various enzymes and structural proteins [45].

Intracellular plant compartments and their relative ratio constitute the macro- and micronutrients in the plant and the dispersion of macronutrients varies from plant to plant, changing during aging with a larger proportion of protein and lipids during early growth [46]. In addition to macronutrients and vitamins, the encapsulated plant cells also contain phytonutrients, such as polyphenols (flavonoids and stilbenes), carotenoids, plant sterols, and poly-unsaturated fatty acids (PUFA), many of which are used as nutraceutical ingredients and exhibit beneficial effects on metabolic and cardiovascular parameters [47].

Although monomers from the cell wall degradation process are absorbed via host transporters, most of the plant-cell wall functions as microbiota substrate [12]. Furthermore, the mixture of macronutrients, vitamins, and phytonutrients encapsulated within the plant cell membrane have a two-tier function, with part of their host-health-promoting effects also benefiting the microbiota. In other words, the microbiota and the host share both host-enzyme-resistant carbohydrates and fiber-encapsulated macro- and micronutrients.

## 4. Microbiota Composition

In human adults, five bacterial phyla have been reported to be dominant in the gut microbiota of healthy individuals: *Firmicutes, Bacteroidetes*, *Actinobacteria, Proteobacteria* and *Verrucomicrobia* [48], with more than 90% of the species belonging to *Firmicutes* and *Bacteroidetes*. Representatives of the other phyla comprise from 2 to 10% with great interpersonal variation [49]. Changes in the relative abundance of the two dominant bacterial divisions, *Bacteroidetes* and *Firmicutes*, have been associated with an increased BMI [50]. An overrepresentation of butyrate-producing species, such as *Roseburia* spp. and *Eubacterium* spp. within the *Firmicutes* phylum affects the metabolic potential of the microbiota, i.e., the microbiotic capacity to harvest energy from the diet and produce SCFA available for host consumption [51].

In humans, an abundance and diversity in polysaccharide-fermenting bacteria, such as species within the *Bacteroidetes* phylum, are inversely related to obesity and other metabolic disorders with an inflammatory component [52,53,54,55]. Members of *Bacteroidetes* express very large numbers of genes that encode microbiotic enzymes and readily can switch between different energy sources in the gut depending on substrate availability. Within the Bacteroidetes phyla, two genera are particularly dominant and mutual antagonistic; *Bacteroides* and *Prevotella* [56]. The relative ratio between these two genera varies, with the *Bacteroides*-driven type reported as dominant in individuals with a higher intake of protein and animal fat, and *Prevotella* more common in individuals who consume more carbohydrate-rich diets [57,58] These findings represent distinct community composition types—so-called enterotypes—based on genus composition [59], suggesting that such compositional differences both reflect dietary intake and determine an individual’s response to different diets [55].

Long-term dietary habits have considerable effect on the gut microbiota as evidence by differences in microbiotic compositions across geographical and/or cultural-dependent diets [28,57,60]. Microbiota composition also respond to short-term macronutrient changes; moreover, functional traits linking enterotypes to diet have been identified [57,58]. Animal and human studies show that an acute dietary switch results in dramatic shifts in the gut microbiota within the course of 24 h [16]. An animal-based diet increases the abundance of bile-tolerant genera (*Alistipes*, *Bilophila*, and *Bacteroides*) and decreases the level of *Firmicutes* species capable of fermenting dietary plant polysaccharides. Microbiotic activity mirrors differences between herbivorous and carnivorous mammals, reflecting trade-offs between carbohydrate and protein fermentation [61]. Analyses of the relative abundance of taxonomic groups have shown that short-term animal-based diets have a greater impact on microbiotic community structure compared to plant-based diets [16], suggesting perhaps a default microbiotic setting favoring plant-based diets.

As mentioned above, bacterial species have varying abilities to degrade fibers from different sources. The ability to degrade a wider variety of complex carbohydrates therefore forms a more diverse bacterial community. Moreover, the biodiversity, function, and abundance of a microbiotic community also depend on cross-feeding among members, which is the process where essential metabolites synthesized by a subset of the community provide substrate for growth of another/other bacterial species [62]. These microbiotic interactions create a complex pattern of interconnected metabolisms and link members of different genera/phyla together in communities.

Biodiversity therefore contributes to the stability and resilience of the bacterial communities. A high level of resilience ensures renewal and reorganization of the community if a species is lost, whereas communities with low biodiversity are more susceptible to loss of individual species. Highly diverse ecosystems most likely contain species with redundant functions, making the loss of a single species tolerable, because other functionally redundant species can take over [63]. Evidently, the microbiotic composition and community organization are reflective of dietary habits and affect host metabolic and immune processes. The distinction between enterotypes with distinct fermentation kinetics should therefore be considered an environmental factor that contributes to both health and disease.

## 5. Biological Journey and Destiny of Complex and Simple Carbohydrates

Monosaccharides can be absorbed via transporters in the human GI tract whereas polysaccharides that are not encapsulated by a fibrous plant cell wall, undergo digestion in the upper GI tract devoid of the microbiota. The more complex the carbohydrate the more glycosidase activity is necessary for the depolymerization. Thus, the more complex the plant cell wall (nutrient delivery device), the more microbiota interaction is needed. As a result, more caloric harvesting takes place and a larger intestinal area is subject to nutrient absorption, enteroendocrine stimulation, and immunological maturation.

The human gut has the potential to absorb monosaccharides via several transporters located in the absorptive epithelium. Apical sodium-dependent glucose cotransporter 1 (SGLT1) [64], glucose transporter 2 (GLUT2) [65], and glucose transporter 5 (GLUT5) [66] facilitate absorption of glucose and fructose from the intestine, and basolateral GLUT2 and GLUT5 facilitate the basolateral monosaccharide secretion into the host interstitium. Upon ingestion of a sugar-enriched meal, a transient upregulation of the absorptive monosaccharide transporters is induced, perhaps via apical recruitment of GLUT2 [67,68,69]. Thus, sugar ingestion promotes host absorption and bypasses caloric harvesting by microbiota.

Throughout the GI tract the level of glucose transporters is highly regulated by hexose availability and insulin levels [70,71,72,73]. In addition, the expression of glucose transporters has been shown to be increased in e.g., obesity and diabetes [74,75] and as a result, certain individuals experience a larger bioavailability of monosaccharides.

Despite individual differences in host digestive enzyme- and nutrient transporter expression, a general notion must be highlighted: The density and diversity of the microbiota increases with the distance from the stomach. The more complex the carbohydrate, the longer the journey until complete fermentation, and therefore the more likely that carbohydrates are utilized by the microbiota and not by the host.

## 6. The Plant Cell Wall Viewed as a “Pharmaceutical-Nutrient Formulation”

When applying the drug delivery perspective to unrefined plant-based foods, it is evident that the polysaccharides of plant-cell walls [76,77] are more than just molecular nutrition for the host and microbiota [78]. Unrefined plant-based food structures can be interpreted as a slow release device for nutrients and bioactive molecules in the human GI tract, which has important implications for intestinal absorption and local biological actions. The “device” releases a complex pool of molecules with nutritional value for the host and the microbiota, and with biological effects that trigger the endocrine and immunological maturation of the host. The release of nutrients from the fiber-encapsulated plant cell is thus dependent on microbiota-specific plant cell wall fermentation.

In pharmaceutical drug discovery and development processes, both pharmacokinetic and pharmacodynamic properties are carefully optimized. Whereas the optimized drug molecule determines the specific pharmacological action, the site of action and the duration of action are heavily influenced by the pharmaceutical formulation of the drug molecule. For example, a specific formulation can be used to target sustained or fast absorption from the GI tract. In other words, the drug molecule needs a relevant formulation to reach the relevant site of absorption and to obtain the desired absorption kinetic [79].

**Figure 2:**
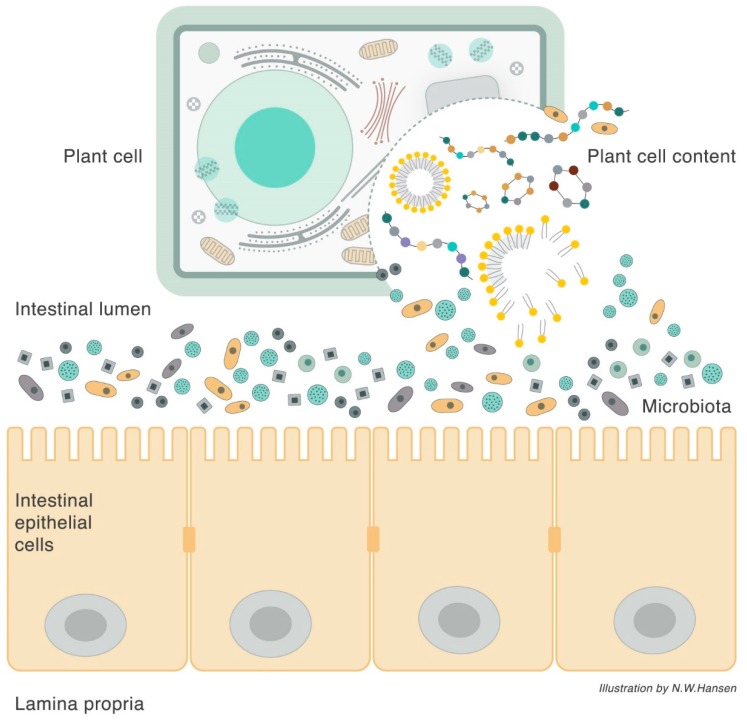
Illustration of interactions between plant cells, microbiota, and the host epithelium. Plant cells are encapsulated by the fibrous cell wall, which is a microbiota-selective nutrient. The cell wall also serves as a transport vehicle for the additional plant cell contents as well as a vehicle for nutrients to be shared between the microbiota and the host upon release from its plant cell structure. The microbiota secretes glycosidases, peptidases, and lipases, thereby releasing molecular nutrients for transport into epithelial or bacterial cells.

Different saccharides (sugars, starches, and fibers) are used as coating material in oral drug formulations to direct drugs through the GI infrastructure and deliver the drug molecule at the desired site of action or absorption [80]. Salicylic acid is, for example, formulated for fast or sustained systemic absorption using different formulation principles. Different cellulose coatings have been used to deliver the drug to distal parts of the gut for local IBD therapy [81,82]. Basically, the microbiota ferments the coating material and releases the drug distally for sustained release (or local treatment). The diversity in fibrous plant cell structures therefore serves as both a saccharide delivery system for bacterial species and as a fiber-encapsulated delivery device of other molecular nutrients (e.g., amino acids, fatty acids, vitamins, and other bioactive molecules). These nutrients are released from the plant cell upon plant-cell-wall fermentation, playing a major role for both host and microbiota biology (Figure 2). Indeed, most bacteria, even *Lactobacillus* and *Bifidobacterium*, express lipid and amino acid transporters [83,84,85,86] and regulate the expression dependent on the specific environment.

The biological properties of the microbiota are not solely dependent on the carbohydrate-based prebiotic content, but indeed also on the content of lipids, proteins, amino acids, and vitamins [87]. In vitro studies of bacteria emphasize the perspective that plant-based nutrients beyond fibers are important for microbiotic function. For example, the growth and adhesion properties of specific microbial strains are different in two different media (soy bean media and MRS media) [88] and the growth of *Lactobacillus* and *Bifidobacterium* depends on the media protein content [89,90]. Therefore, a diversity of media is needed to actually culture a diversity of human gut microbiota and study their selective and community properties [91]. Peptides and lipids that are not fiber-encapsulated would most likely only reach the microbiota in limited amounts due to upper-intestinal host absorption. Thus, a major source of amino acids and lipids for the microbiota is formulated in plant cells.

If plant cells have been refined, this organization does not remain intact, and even if the fibers reach the microbiota (e.g., fiber supplements), only limited amounts of amino acids and lipids will reach the microbes.

Some bacterial genera e.g., *Bacteroides* [92] and *Lactobacillus* [93] secrete both peptidases and glycosidases and the more distal fiber-fermenting species *Bacteroides thetaiotaomicron* also express dipeptidylpeptidases (DPPs) [94,95]. Interestingly, the DPP4 antagonist vildagliptin, used for the treatment of T2D, induces a higher relative abundance of *Bacteroidetes*, a lower abundance of *Firmicutes*, and thus a reduced ratio of *Firmicutes/Bacteroidetes* in rats [96]. This is one among many examples of anti-diabetic drugs that has recently been shown to induce secondary health promoting actions via the microbiota [97].

Another pharmaceutical exploration of the microbiota is the study of bacterial peptide transporters with the aim to identify new antibiotic targets in virulent bacteria. Since blockage of peptide transporters is a potent antibiotic pathway, specific bacteria are dependent on their endogenous peptide transporters and on amino acid fuel [98]. In both gram negative and gram positive virulent bacteria, two distinct vital types of peptide transporters exist, proton-dependent peptide transporters (PRT or POT) [99] and ATP-binding cassettes (ABC transporters) [100].

As in the case of amino acids, energy dependent fatty acid transporters are also present in both outer and inner membranes of bacterial strains. Even *Lactobacillus* expresses amino acid and fatty acid transporters [83], which supports the bacterial ability and need to consume fatty acids slowly released from plant cells during the microbiotic digestion of the plant cell wall. In specific bacteria, the process requires the outer membrane-bound fatty acid transport protein FadL and the inner membrane associated fatty acyl CoA synthetase (FACS) [101,102].

The diversity in fiber-coated plant cell structures also serves as delivery system for local acting biologically active substances, e.g., taste receptor agonist for distal enterocytes and the microbiota [103], and maturation agents for the immune system [104] which will be discussed below.

## 7. Pro-Inflammatory Effects of Refined Foods

The consumption of unrefined plant-based foods maintains host health by engaging the microbiota. The immunomodulatory effects of unrefined plant-based foods act through the host-microbiota symbiosis and circumvent the pro-inflammatory effects of refined foods. Mono- and disaccharides in a fibrous sustained release formulation do not produce significant increase in blood glucose because they are partially consumed by the microbiota. The microbiota unwraps molecules from the fibrous formulation in a time-consuming process. In contrast, industrial refinement causes a lack of molecular engagement with the microbiota and distal enterocytes, and polysaccharide molecules are rapidly monomerized and absorbed with the risk of elevated blood glucose levels [26].

Mounting epidemiological evidence suggests that intake of refined sugars and starch is associated with the development and maintenance of several diseases. T2D, for example, is driven by insulin resistance and β-cell dysfunction and manifested by increased risk and severity of hyperglycemic events. The condition is associated with elevated risk of autoimmune and inflammatory diseases, including rheumatoid arthritis [105] asthma [106], psoriasis [107], and cancer [108,109]. Drugs or dietary regiments that normalize hyperglycemia do not surprisingly have a therapeutic effect on immune diseases, e.g., psoriasis [110,111,112] and rheumatoid arthritis [113].

For diabetics as well as healthy individuals there is a relationship between the ingestion of refined sugars and the risk of developing both obesity [114] and autoimmune/inflammatory diseases, e.g., rheumatoid arthritis [115], asthma [116,117], IBD [118], chronic kidney disease [119], and atherosclerosis [114,120].

Hyperglycemia influences inflammation and immune function through multiple mechanisms. Chemical reactivity of glucose and other reducing sugars (fructose, mannose, galactose) towards proteins [121], lipids, and nucleic acids [122] is important. Non-specific stochastic protein glycations during incidents of hyperglycemia have the potential to initiate and potentiate several inflammatory and immunomodulatory responses, involving activation of pattern recognition receptors (PRR), e.g., RAGE (receptors for advanced glycation end-products) [123,124]. Also, hyperglycemia-induced cytokine release [125], formation of methylglyoxal and other auto-oxidation metabolites, e.g., reactive oxygen species (ROS) and free radicals [126] maintains inflammatory responses. The activity of auto-oxidation metabolites is prevented, for example, by antioxidants, which reduce the oxidative damage induced by glucose metabolites and ROS [127]. The fact that antioxidants are prevalent in plant-based food and synthesized by the microbiota [128] adds to the anti-inflammatory effects of unrefined foods.

## 8. Anti-Inflammatory Effects of Unrefined Foods

The anti-inflammatory effects of unrefined foods are particularly mediated by their contribution to a high gut biodiversity, which as mentioned above, contributes to the stability and resilience of the bacterial communities. In contrast, the loss of diversity can cause an imbalance between the inflammatory and the immunoregulatory taxa present in a given microbiota. The effects of a complex microbiotic community are ascribed to the additive and synergistic effects of the bacterial species in a microbiotic community. An example of this is monocolonization of a variety of organisms, which cannot reverse the hyper-IgE phenotype in germ-free (GF) mice. However, complex polycolonizations restore normal IgE levels [129].

Imbalances in immunological homeostasis can occur due to the loss of habitat-specific species and symbionts, resulting in an invasion of opportunistic species not normally able to colonize that specific habitat within the intestinal mucosa. Such a mismatch between species and habitat triggers a potentially pathogenic host response [130]. The detection of pathogens by the host is achieved through the families of PRR localized in the intestinal epithelium. PRR recognize conserved molecular structures known as microbe-associated molecular patterns and induce production of innate effector molecules [131,132]. The most well-known PRR are the toll-like receptors (TLR), RAGE, and the NOD-like receptors expressed on the intestinal epithelial cells. Activation of TLR by bacterial products promotes epithelial cell proliferation, secretion of IgA into the gut lumen, and expression of antimicrobial peptides, thereby establishing a microorganism-induced system of epithelial cell homeostasis and repair in the intestine [132].

Bacterial translocation, e.g., infiltration of lipopolysaccharide (LPS) through the intestinal barrier, can promote local and systemic immune responses via PRR. Monocolonization of GF mice by *Escherichia coli* is, for example, sufficient to induce macrophage infiltration, and polarization towards pro-inflammatory phenotype of immune cells in the adipose tissue [133], contributing to a state of low-grade inflammation in the adipose tissue. Disruption of intestinal barrier integrity by viable bacteria has been attributed to various intestinal inflammatory diseases such as IBD, celiac disease, irritable bowel syndrome, and colorectal cancer [32] as well as to diseases in extra-intestinal organs, such as the liver [134].

The major microbiota-derived SCFA, butyrate, acetate, and propionate are implicated in multiple anti-inflammatory mechanisms [135]. Besides being used by colonocytes as a source of ATP, SCFA can act as extracellular signaling molecules that activate cell-surface free fatty acids receptors (FFAR) (G-protein-coupled receptors (GPCR)) or inhibit histone deacetylases (HDAC) [136]. FFAR are differentially expressed on adipocytes, immune cells, and enteroendocrine L-cells. Depending on the location of the receptor and the amount and type of SCFA, the response can have multiple, variable downstream effects [3]. FFAR, expressed on immune cells, such as macrophages, dendritic cells, and neutrophils will upon activation by butyrate inhibit the release of pro-inflammatory cytokines, or stimulate differentiation of T regulatory (Treg) cells [136].

Activation of FFAR2/GPR43 by butyrate on enteroendocrine L-cells of the ileum and colon induces production and secretion of intestinal peptide YY (PYY), glucagon-like peptide 1 (GLP-1) [25,137]. An abundant production of SCFA can therefore indirectly regulate blood glucose levels via the insulinotropic effect of GLP-1 and induce satiety by the anorexigenic effect of PYY on the hypothalamus [138]. In addition, enteroendocrine cells in the duodenum are dependent on microbe-mediated mechanisms to express cholecystokinin (CKK), a gut peptide hormone responsible for stimulating fat absorption. GF mice have exhibited a reduced number of these cells with impact on their fat absorption [139].

SCFA can also act by inhibition of histone deacetylase (HDAC) activity. HDAC is related to a suppression of malignant transformation and a stimulation of apoptosis of precancerous colonic cells [140] as well as to the stimulation of epithelial production of retinoic acid (RA) [141]. RA is involved in many physiological processes, including regulation of IgA [142] and the polarization of specialized mucosal myeloid cells [143].

Specific phytonutrients entail several immunomodulatory functions. Some are mediated by a shift in microbiotic composition favoring the abundance of specific bacteria with health promoting effects or create occupancy resistance to enteric pathogens with pro-inflammatory potential [144]. Others are receptor-mediated, e.g., genistein, a flavonoid compound present in legumes, which can positively effect β-cell mass and mitigate T2D in mice [145,146].

In summary, the biodiversity of the microbiotic community is critical for both inter-microbiotic interactions and host-microbe engagements. In a healthy and proper fed microbiota the inflammatory and immunoregulatory species are in balance and the diverse bacterial communities act together to produce metabolites with direct effect on host health.

## 9. The Interaction between Food, Microbiota, and Host

In the sections above, we have discussed how ingestion of unrefined plant-based food structures implies that the host shares calories, saccharides, amino acids, fatty acids, and bioactive molecules with the microbiota, which in return shares SCFA, vitamins, antioxidants, and other biomolecules with the host. The microbiota also stimulates mucosal immune function upon ingesting an unrefined diet; a diverse microbiota will express a multitude of different antigens, thereby promoting intestinal bacterial recognition and shaping intestinal immune function.

**Figure 3:**
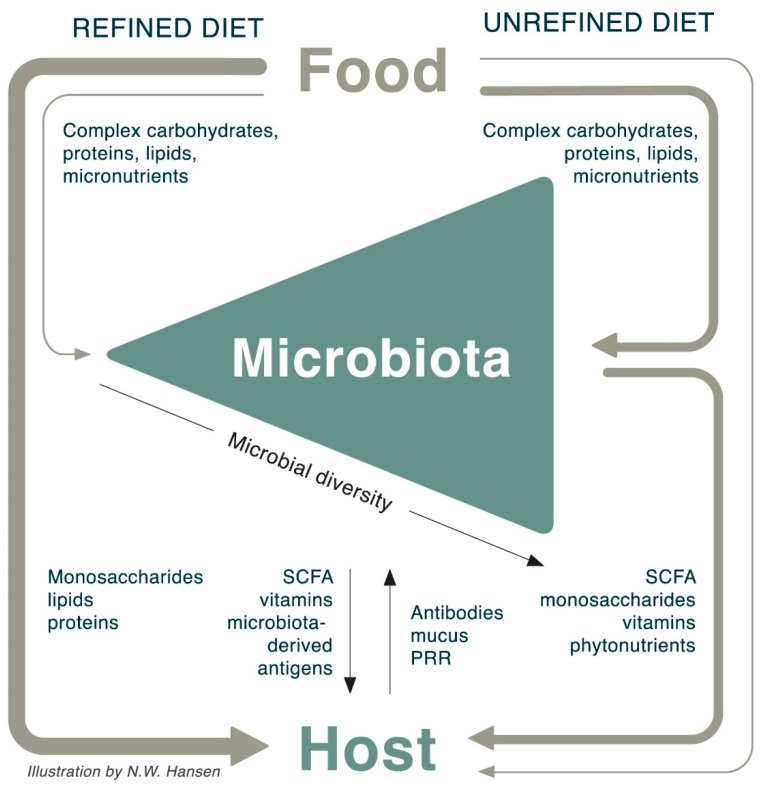
Schematic overview of interactions between food, microbiota, and host. Most of a refined diet bypasses the microbiota, providing a high nutrient bioavailability for the host. In contrast, unrefined, plant-based food structures provide a lower nutrient bioavailability for the host, using the difference to develop and maintain a healthy microbiota. The healthy microbiota provides short chain fatty acids (SCFA), antioxidants, and vitamins for the host along with direct immunomodulatory effects. The high monosaccharide bioavailability in refined foods introduces a risk of hyperglycemia, which cause numerous direct and indirect pro-inflammatory actions [26] (not shown in figure).

In contrast, refined foods lack the fiber-encapsulation with the result that the host absorbs most nutrients in a microbiota-independent manner. Refined food is thus directly absorbed through the host GI mucosa without feeding the microbiota and without immunological engagement in the distal gut (Figure 3). Furthermore, when bypassing the microbiota, glucose is rapidly absorbed creating a risk of high blood glucose and associated pro-inflammatory effects.

## 10. Implications for Nutritional Guidelines

The mechanisms summarized above underscore the need to consider the microbiota when evaluating human development, nutritional needs, physiological variations, and the impact of diet. Historically, the processing of foods occurred to increase the caloric bioavailability but given the growing prevalence of non-communicable diseases, insulin resistance, overweight, and obesity [147], it is obvious that current nutritional guidelines need to be revisited to re-establish the endogenous microbiota-induced health promotion. The current nutritional guidelines are mainly based on optimal molecular nutrient content for host metabolism, while establishment of synergies between food, host, and microbiotic community are absent.

Nutritional guidelines need to consider both the content and the structure of carbohydrates, because the microbiotic breakdown of carbohydrates strengthens our microbiotic ally in support of host health. Furthermore, it should also reflect that consumption of “fiber-formulated nutrients” is essential to maintain a healthy diverse microbiota to balance our nutritional, immunological, and metabolic health.

**Figure 4:**
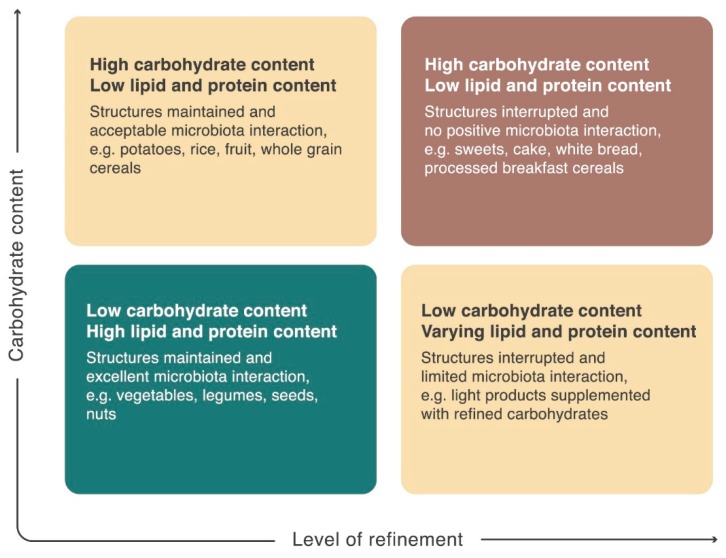
Novel classification of plant-based foods. Plant-based nutrition classified by microbiotic interaction potential and by the potential to induce hyperglycemia. The illustration classifies plant-based foods by the level of refinement and carbohydrate content. The green box represents basic foods for the habitual diet, interacting with our gut microbiota and providing both nutrients and bioactive molecules to microbiota and host. Foods from the green box are health promoting through interaction with the microbiota, and these foods introduce no risk of hyperglycemia. Foods from the green box should be combined to meet the needs for protein and lipid. Food from the yellow boxes can induce hyperglycemia in insulin resistant individuals or be consumed as energy supplementation for children and physically active individuals with a higher energy demand and a low risk of insulin resistance. The yellow square to the left positively interacts with the microbiota whereas the yellow square to the right only have limited microbiota interaction. The red box contains foods, which are high in carbohydrate content and highly refined, introducing the risk of high blood glucose to most people, not only insulin resistant individuals. The contents in the red box should be considered comfort foods with no health promoting effects and with a potential to compromise health.

We have developed a simple tool to visualize healthy microbiota-engaging food choices (Figure 4). This tool divides foods into four categories depending on their carbohydrate content and the degree of refinement. Healthy, unrefined foods with low relative carbohydrate content (green box) should make up the plant-based bulk of an adult basic diet. These foods interact with the microbiota. Refined foods or foods with high relative carbohydrate content (yellow boxes) should be considered carbohydrate or energy supplementation, while the “comfort” foods in the red box, which are both refined and high in carbohydrate content, should be used sparingly considering their potentially negative health impact and their lack of microbiotic health promotion.

## 11. Conclusions

The increasing prevalence of non-communicable diseases with an inflammatory component has led to the understanding of the gut microbiota as an intrinsic regulator of host immune responses. There is a growing awareness of the role of microbiotic communities, microbiota-derived products, and more particularly of the link between these components and disease states in humans. The ability to target immune pathways relies on the understanding of both acute and long-term impacts on the gut microbiota. The microbiotic communities can adapt to host diet in multiple ways by the interaction among different bacterial species, loss or proliferation of specific species, and by interaction with the host. Diet therefore presents itself as a microbiota-modulating factor and hence a health-modulating factor. A revision of the current nutritional guidelines considering both the molecular content and molecular structure of nutrients for insulin resistant and insulin sensitive individuals, could be valuable for the restoration of beneficial bacteria and microbiota diversity, enabling a shift from disease to health promoting states in the individual as well as in the general population.
